# Supplementary material for: Bioactive Variability and In Vitro and In Vivo Antioxidant Activity of Unprocessed and Processed Flour of Nine Cultivars of Australian lupin Species: A Comprehensive Substantiation
Source: Antioxidants (Basel). 2020 Mar 27;9(4):282. doi: 10.3390/antiox9040282 (PMC7222189; doi:10.3390/antiox9040282)
Supplement: Supplementary file 1 [file antioxidants-09-00282-s001.pdf]

Supplementary File

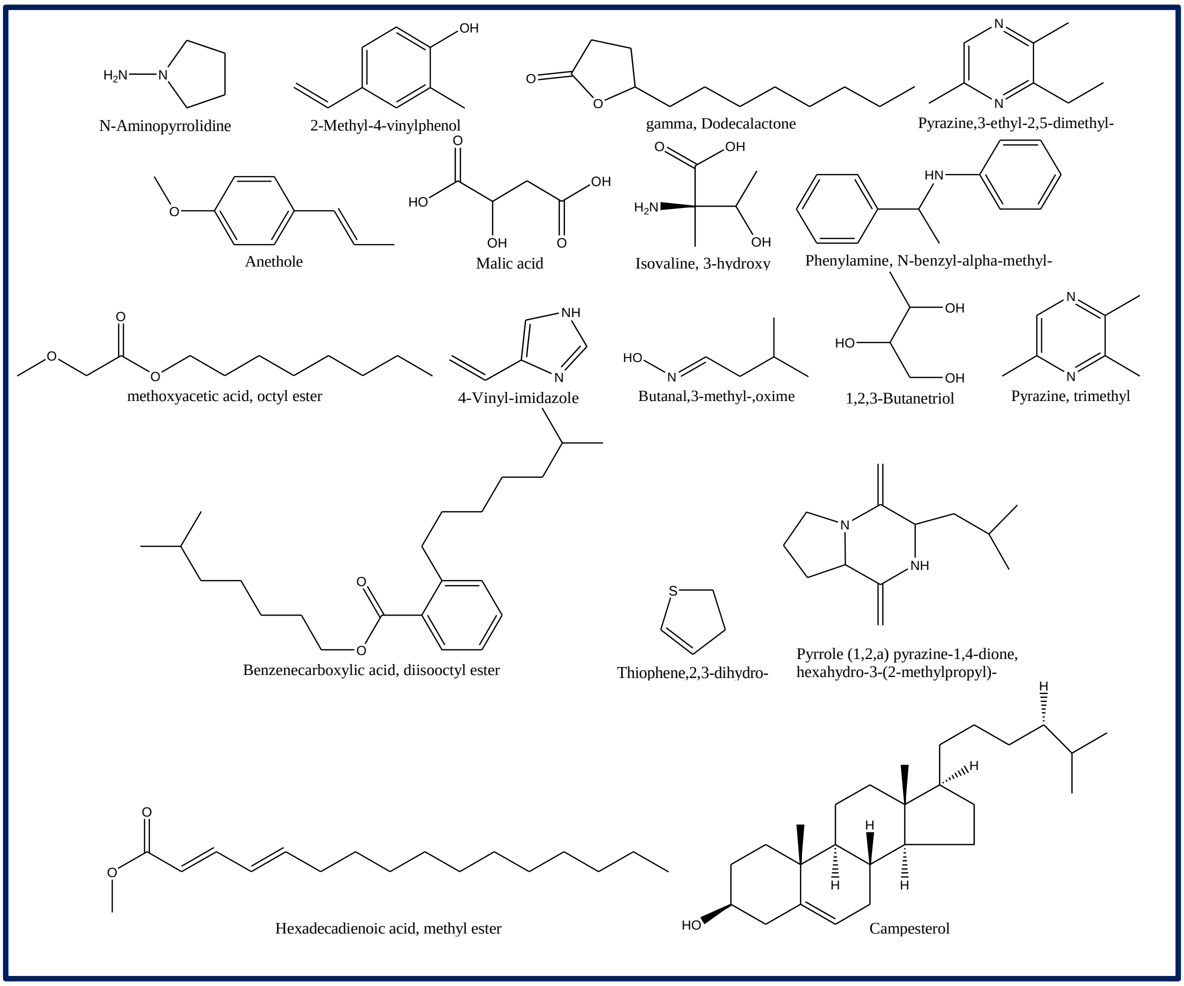

Figure S1. Structure of bioactive compounds present in lupin species.

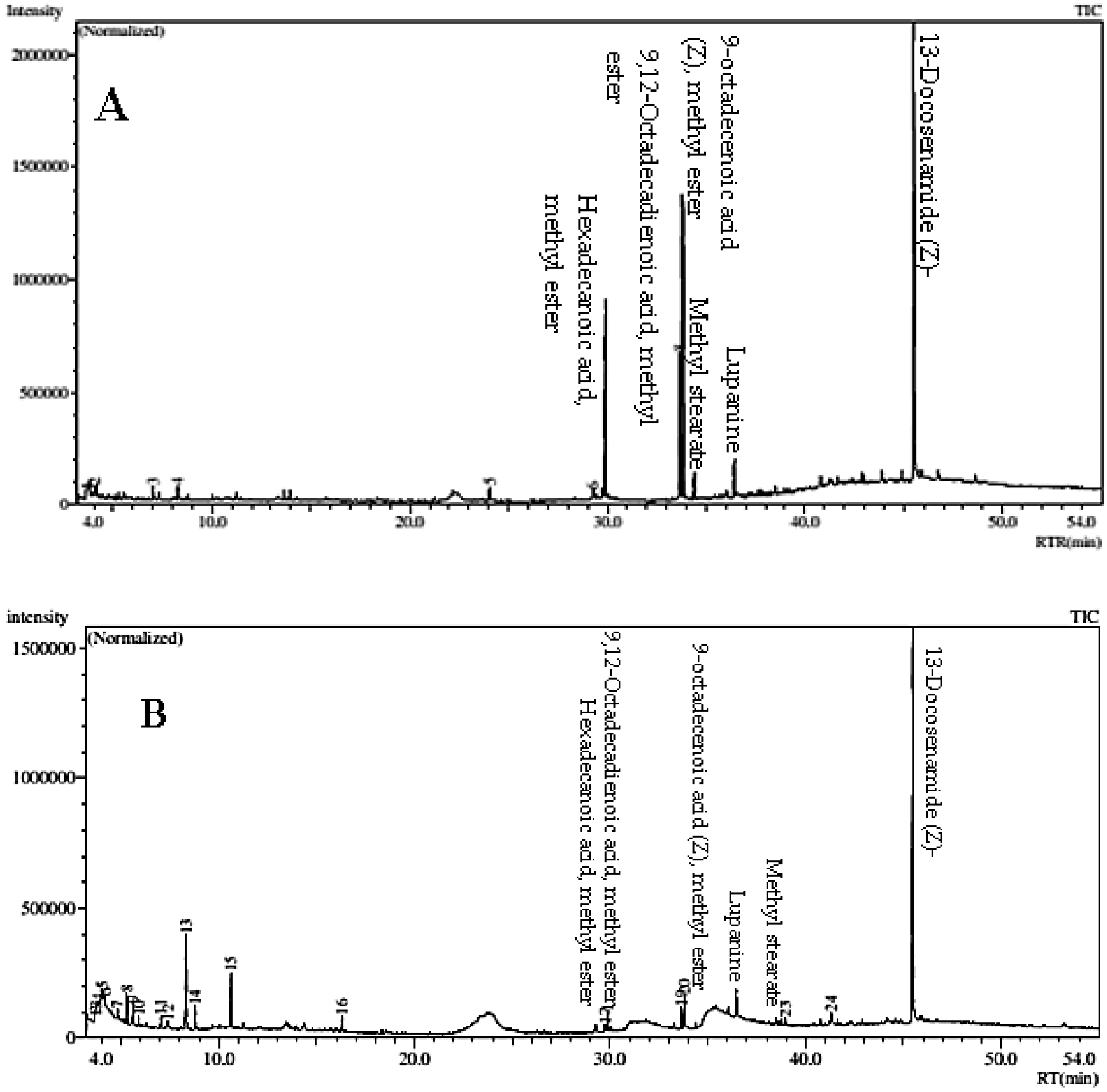

Figure S2. Common six compounds identified in unprocessed (A) and processed (B) flours of Lupin cultivar Jenabillup (JEN) in GC-MS analysis.

Table S1. Pharmacological activities of bioactive compounds present in lupin species.

Compound class	Bioactive compounds	Retention time (min)	m/z	Pharmacological activity	Ref
Quinolizidine alkaloid	Lupanine	36.44	136.00	Inhibition of lipid peroxidation and free radical scavenging, antidiabetic activities	[28,29]
	13-OH-Lupanine	41.29	152.00	Antioxidant activity	[31]
	13a-Acetoxylupanine	46.56	246.00	Antifungal, antibacterial activities	[34]
Fatty acid methyl ester	Hexadecanoic acid, methyl ester	33.64	67.00	Antifungal and antioxidant activities	[24]
	9,12-Octadecadienoic acid, methyl ester	33.64	67.00	Antioxidant and antimicrobial activities	[26]
	11-Octadecenoic acid, methyl ester	33.82	55.00	Antioxidant and antimicrobial activities	[26]
	Methyl sterate	34.40	74.00	Antioxidant activity	[27]
	7,10-Hexadecadienoic acid, methyl ester	38.68	67.00	Antioxidant, anti-inflammatory, hypocholesterolemic and cancer prevention activities	[30]
	9-Octadecenoic acid, methyl ester	33.83	55.00	Antioxidant activity	[26]
	Methyl tetradecanoate	24.05	74.00	Antifungal and antioxidant activities	[38]
	Dodecanoic acid, methyl ester	16.72	74.00	Antioxidant activity	[39]
	9-Hexadecenoic acid, methyl ester, (Z)-	33.80	55.00	Antioxidant activity	[26]
	1-Monolinoleoylglycerol Trimethylsilyl ether	38.52	73.00	Antioxidant, anticancer and antimicrobial activities	[48]
	Triacontanoic acid, methyl ester	41.67	149.00	Antioxidant activity	[49]
	Oleic acid, 3-hydroxypropyl ester	41.77	55.00	Antioxidant activity	[51]
	Methyl tetradecanoate	24.05	74.00	Antioxidant activity	[53]
	Methyl 9-cis,11-trans-octadecadienoate	34.73	67.00	Antioxidant activity	[54]
Volatile oil	Anethole	10.59	148.00	Antioxidant activity	[19]
Sterol	Campesterol	53.35	55.00	Antioxidant activity	[35]
Chromo-peptide	Actinomycin C2	29.49	154.00	Antimicrobial, antioxidant and anticancer activities	[23]
Amine	N-Aminopyrrolidine	8.42	85.00	Antioxidant activity	[17]
	13-Docosenamide, (Z)-	45.47	59.00	Antioxidant and antimicrobial activities	[33]
Heterocyclic compound	Pyrazine, 3-ethyl-2,5-dimethyl-	5.55	135.00	Antioxidant activity	[15]
	Pyrazine, trimethyl	4.186	122.00	Antioxidant activity	[15]
	Pyrazine, tetramethyl-	5.73	54.00	Antioxidant activity	[15]
	2-Pyrrolidione	5.94	85.00	Antioxidant activity	[17]
	Thiophene, 2,3-dihydro-	8.36	85.00	Antioxidant activity	[17]
	4-Vinyl-imidazole	8.53	94.00	Antioxidant activity	[18]
	pyrrole (1, 2, a) pyrazine 1, 4, dione, hexahydro 3-(2-methyl propyl)	29.77	70.00	Anticancer and anti-cholinesterase activities	[22]
	2,4-Dihydroxy-2,5-dimethyl-3(2H)-furan-3-o	3.82	101.00	Antioxidant activity	[43]
	2-Piperidinone	5.68	85.00	Antioxidant activity	[44]
	4H-Pyran-4-one, 2,3-dihydro-3,5-dihydroxy	4.82	71.00	Antioxidant activity	[52]

Lactone	gamma, Dodecalactone	22.21	85.00	Antioxidant activity	[21]
Polyunsaturated hydrocarbon	Squalene	45.97	69.00	Antioxidant activity	[46]
Volatile compound	2,4-Decadienal, (E,E)-	11.42	81.00	Nematicidal Activity	[47]
Indole	(-)-Indole-2-one, 2,3-dihydro-3-[2-[N-dimeth	16.32	58.00	Antioxidant activity	[50]
Alcohol	1,2,3-Butanetriol	5.39	45.00	Antioxidant activity	[16]
	2-Methyl-4-vinylphenol	11.21	150.00	Antioxidant activity	[20]
	2-Methoxy-4-vinylphenol			Antioxidant activity	[45]
Carboxylic acid	Malic Acid	5.289	89.00	Antioxidant activity	[37]
Others	5,10-Diethoxy-2,3,7,8-tetrahydro-1H, 6H	30.00	70.00	Antioxidant activity	[25]
	1,2-Benzenecarboxylic acid, diisooctyl ester	41.67	149.00	Antioxidant activity	[32]
	Methoxyacetic acid, octyl ester	4.02	45.00	Antioxidant activity	[36]
	Divinyl sulfide	3.27	85.00	Antioxidant and antinociceptive activities	[42]
	Benzene, 1-methoxy-4-(1-propenyl)-	10.60	148.00	Antioxidant, anti-inflammatory and gastroprotector activities	[44]
	Butanal,3-methyl-,oxime	4.33	59.00	Antioxidant activity	[16]
	Di-n-octyl phthalate	41.67	149.00	Antioxidant activity	[55]

Table S2. Bioactive molecules obtained by GC-MS analysis in nine cultivars of processed and unprocessed lupin flours.

Compounds name	Concentration (%)																	
	BAR		GUN		JEN		JIN		MAN		LUX		ROS		WK		JUR	
	Un	P	Un	P	Un	P	Un	P	Un	P	Un	P	Un	P	Un	P	Un	P
Pyrazine, trimethyl				1.01		1.12	0.93			1.13		1.07						
Butanal,3-methyl-,oxime									12.30	8.97		23.04						
1-Propanol, 2-(2-hydroxypropoxy)-											0.19	0.99						
1,2,3-Butanetriol	2.44										1.43	2.24						
Pyrazine, 3-ethyl-2,5-dimethyl-									0.29			0.32						
Methoxyacetic acid, 2-methylpropyl ester									0.92			1.45		1.23				
Pyrazine, tetramethyl-											0.92	1.63						
2-Pyrrolidione							6.03	7.44				5.15		2.22		5.78		
Thiophene, 2,3-dihydro-						16.81	0.83	0.58	1.23	2.34		1.27	1.29					
N-Aminopyrrolidine											4.31	2.12	1.22					
4-Vinyl-imidazole												0.78	0.82	0.27				
Anethole				4.29		6.13		1.17	2.00			1.07				2.78		
2-Methyl-4-vinylphenol											1.92	1.18	3.09	2.18				
N-Hydroxymethyl-2-phenylacetamide									1.97			1.85						
Benzeneethanol, 4-hydroxy-			11.30								2.81	2.41						
Phenol, 2,4-bis(1,1-dimethylethyl)-									1.22	0.22		2.18						
Cyclohexane, pentyl-										1.23		1.32						
2(3H)-Furanone, dihydro-5-(2-octenyl)-, (Z)-							6.53	6.91	2.18			2.25						
Gamma, Dodecalactone							2.90	2.76	0.83		2.22	1.28						
3-Methyl-1,4-diazabicyclo(4.3.0)nonan-2,5-d										1.22	0.99	1.18						

Pyrrole (1, 2, a) pyrazine 1, 4, dione, hexahydro 3-(2-methyl propyl)				0.92	1.00	0.78	1.06	1.01	1.93			0.86						0.72
Actinomycin C2										0.68		2.33						
Hexadecanoic acid, methyl ester	31.06	5.73	8.52	6.28	25.92	2.87	2.29	1.32	18.30	2.89	18.22	2.67	0.23	0.11	10.08	3.29	2.91	20.70
5,10-Diethoxy-2,3,7,8-tetrahydro-1H, 6H												2.28						
Di(cyclopentanoyal-2)methane										1.55		0.5			1.46			
2-Pentylcyclopentanone												1.76						
9,12-Octadecadienoic acid, methyl ester	4.64	2.22	4.01	1.45	7.52	1.58	0.59	0.35	11.73	4.82	0.23	0.59	2.12	0.22	3.96	1.24	2.09	3.91
11-Octadecenoic acid, methyl ester	1.07	0.33	0.95	0.35	0.78	0.35	1.21	0.65	0.84	1.22	0.74	1.76	2.13	0.94	0.54	3.88	2.31	0.25
Phenylamine, N-benzyl-.alpha.-methyl-												0.38						
Methyl sterate	3.95	4.39	1.12	1.42	2.93	4.09	3.39	4.29	3.97	0.73	10.47	1.28	5.22	2.39	8.91	0.53	1.44	2.79
Lupanine	1.83	3.58	1.47	1.01	2.73	3.30	3.68	1.70	0.72	0.28	0.24	7.08	2.30	0.12	6.36	0.75	3.59	4.91
9-Octadecanamide									21.90			0.77						
7,14-Methano-4H,6H-dipyridol												0.21	0.11					
Ergotaman												1.05						
7,10-Hexadecadienoic acid, methyl ester									1.67			1.33	0.23					
9-Octadecenamide				0.54			0.36		1.06			0.45						
Ergotamine												0.88		0.21				
13-OH-Lupanine				0.64					1.21	0.81			0.19					
1,2-Benzenecarboxylic acid, diisooctyl ester												0.35						
13-Docosenamide, (Z)-	20.03	48.4	16.87	21.73	31.74	37.42	13.60	12.84	23.21	2.98	13.18	16.43	1.66	3.22	18.33	38.25	18.82	26.44

13a-Acetoxylupanine									9.22	1.88			0.32	0.11				
Campesterol				1.23									0.88					
Methoxyacetic acid, octyl ester										0.13								
Isovaline, 3-hydroxy	0.77								1.82									
Ethylene glycol monoisobutyl ether	3.01																	
Malic Acid	2.17			1.48		3.34	2.26	0.79					3.22			1.33		0.94
9-Octadecenoic acid, methyl ester	11.44	3.34	7.54	5.99	14.09	2.18			15.64	0.09	0.28		5.31	2.34	23.87	0.55	2.19	12.68
Nonanoic acid, 9-oxo-, methyl ester	0.58		0.37	0.32					0.39									
Benzeneethanol, 4-hydroxy-	12.32																	
Methyl tetradecanoate	3.49																	
Dodecanoic acid, methyl ester	1.06																	
9-Hexadecenoic acid, methyl ester, (Z)-	1.20			0.63					0.11		0.17		0.20					
1,2,3,4-Butanetetrol, [S-(R*,R*)]-		4.51																
3,3-Dimethylbutane-2-ol		0.85											0.21	0.03				
dl-Mevalonic acid lactone		1.35							0.31									
cis-Methyl 11-eicosenoate			1.82															
Tetrapentacontane, 1,54-dibromo-			0.66							0.71			0.21					
13-Docosenoic acid, methyl ester, (Z)-			7.20	4.21					0.88		0.27		0.13	0.32				
Citronellol epoxide (R or S)			0.34							0.12			0.05					
2-Nonanol						7.09								0.23				
Divinyl sulfide						0.45												
dl-Glyceraldehyde						9.78												
2,4-Dihydroxy-2,5-dimethyl-3(2H)-furan-3-o						0.36												
Methoxyacetic acid, octyl ester						5.97												

2(3H)-Furanone, dihydro-3-hydroxy-4,4-dimethyl			1.90								0.42				0.60
Hexane-1,3,4-triol, 3,5-dimethyl-			2.31				1.82								
2-Amino-3-methyl-4-pentynoic acid			0.66												
N-Aminopyrrolidine			3.83												
1,4-Dioxane, 2,3-dimethoxy-				0.73							0.35				
1,3-Dioxolane, 2-methyl-				3.03			0.01								
2-Piperidinone				41.88	53.85										
4-Methylenebicyclo[4.2.0]oct-2-ene				1.36	1.30										
Phenol, 3,5-bis(1,1-dimethylethyl)-				1.04											0.41
L-Proline, N-valeryl-, heptadecyl ester	0.83			1.31		0.12									
Dodecanamide, N-isobutyl-				1.36	1.29										
N-Decanoylmorpholine				0.39	0.37					0.24					
4-Nitrobenzoylmethyl-.beta.-phenylpropionat				1.24	1.60										
N-Isobutyl-(6Z,8E)-decadienamide					0.25		0.11								
Hexanoic acid										2.99	0.12				0.81
dl-Malic disodium salt															0.30
Divinyl sulfide		2.02									0.21				0.93
Benzene, 1-methoxy-4-(1-propenyl)-															5.41
2-Methoxy-4-vinylphenol															0.46
1,2-Butanediol, 1-(2-furyl)-3-methyl-															0.47
Hydroxylupanine	0.31					0.64	0.02	0.99	0.13	0.72	0.08		0.09		0.18
Di-n-octyl phthalate															9.35
Squalene	1.05					0.12								1.17	0.93
Glycerin		4.81				28.09						20.10			
2,4-Decadienal, (E,E)-								0.63							
Nonanoic acid, 9-oxo-, methyl ester								1.44							

Cyclopropaneoctanoic acid, 2-[[2-[(2-ethylcy								0.24							
Eicosanoic acid, methyl ester	0.52					2.09		0.89							
Methyl 6-cis,9-cis,11-trans-octadecatrienoate							0.08	0.332							
1-Monolinoleoylglycerol trimethylsilyl ether								0.23							
Naphthalene, decahydro-1,5-dimethyl-								0.68							
Triacontanoic acid, methyl ester								0.17							
2-Hydroxy-gamma-butyrolactone							3.22					5.15			
13-Hydroxy-lupanine TMS derivative	0.22					0.03						0.60	0.56		
3-Allyloxy-1,2 propanediol													2.62		
1,3-Butanediol, 2-methyl-													3.38		
(-)-Indole-2-one, 2,3-dihydro-3-[2-[N-dimeth													3.66		
Pyridine, 1-acetyl-1,2,3,4-tetrahydro-5-(2-pip													0.30		
Di(cyclopentanonyl-2)methane													2.08		
Alpha-Isolupanine	0.74					18.31							15.45		
Oleic acid, 3-hydroxypropyl ester	0.72					1.01							3.65		
cis-13,16-Docasadienoic acid							0.91						1.89		
Methanone, dicyclopropyl-		3.58									0.15				
4H-Pyran-4-one, 2,3-dihydro-3,5-dihydroxy		1.34	1.39												
Methyl tetradecanoate		2.34									0.12				
1,4-Dioxane, 2,3-dimethoxy-				0.73	0.79					1.3<br>8					
Methyl 9-cis,11-trans-octadecadienoate				0.46	0.60										
N-Decanoylmorpholine				0.39	0.37		0.23								
Hexadecanoic acid, 2-hydroxy-1-(hydroxymet	0.21					1.03								1.03	0.40

Di-n-octyl phthalate	0.1<br>3	0.85	9.35

Reference

1. Song, F.-L.; Gan, R.-Y.; Zhang, Y.; Xiao, Q.; Kuang, L.; Li, H.-B. Total phenolic contents and antioxidant capacities of selected chinese medicinal plants. *Int J Mol Sci* **2010**, *11*, 2362-2372, doi:10.3390/ijms11062362.
2. Fernando, C.D.; Soysa, P. Total phenolic, flavonoid contents, in-vitro antioxidant activities and hepatoprotective effect of aqueous leaf extract of Atalantia ceylanica. *BMC Complement Altern Med* **2014**, *14*, 395-395, doi:10.1186/1472-6882-14-395.
3. Almaraz, N.; Campos, M.; Ávila-Reyes, J.; Naranjo-Jiménez, N.; Corral, J.; Gonzalez, L. Antioxidant activity of polyphenolic extract of monofloral honeybee-collected pollen from mesquite (Prosopis juliflora, Leguminosae). *Journal of Food Composition and Analysis - J FOOD COMPOS ANAL* **2007**, *20*, 119-124, doi:10.1016/j.jfca.2006.08.001.
4. Dontha, S. A REVIEW ON ANTIOXIDANT METHODS. *Asian Journal of Pharmaceutical and Clinical Research* **2016**, *9*, doi:10.22159/ajpcr.2016.v9s2.13092.
5. Sahoo, S.; Ghosh, G.; Das, D.; Nayak, S. Phytochemical investigation and in vitro antioxidant activity of an indigenous medicinal plant Alpinia nigra B.L. Burtt. *Asian Pac J Trop Biomed* **2013**, *3*, 871-876, doi:10.1016/S2221-1691(13)60171-9.
6. Ohkawa, H.; Ohishi, N.; Yagi, K. Assay for lipid peroxides in animal tissues by thiobarbituric acid reaction. *Analytical biochemistry* **1979**, *95*, 351-358, doi:10.1016/0003-2697(79)90738-3.
7. Hill, S.; Lamberson, C.R.; Xu, L.; To, R.; Tsui, H.S.; Shmanai, V.V.; Bekish, A.V.; Awad, A.M.; Marbois, B.N.; Cantor, C.R., et al. Small amounts of isotope-reinforced polyunsaturated fatty acids suppress lipid autoxidation. *Free radical biology & medicine* **2012**, *53*, 893-906, doi:10.1016/j.freeradbiomed.2012.06.004.
8. Srinivasan, R.; Chandrasekar, M.J.; Nanjan, M.J.; Suresh, B. Antioxidant activity of Caesalpinia digyna root. *Journal of ethnopharmacology* **2007**, *113*, 284-291, doi:10.1016/j.jep.2007.06.006.
9. Shirwaikar, A.; Rajendran, K.; Kumar, C.D. In vitro antioxidant studies of Annona squamosa Linn. leaves. *Indian journal of experimental biology* **2004**, *42*, 803-807.
10. Jayaprakasha, G.K.; Jaganmohan Rao, L.; Sakariah, K.K. Antioxidant activities of flavidin in different in vitro model systems. *Bioorganic & medicinal chemistry* **2004**, *12*, 5141-5146, doi:10.1016/j.bmc.2004.07.028.
11. Ebrahimzadeh, M.A.; Nabavi, S.F.; Nabavi, S.M.; Eslami, B. Antihemolytic and antioxidant activities of Allium paradoxum. *Central European Journal of Biology* **2010**, *5*, 338-345, doi:10.2478/s11535-010-0013-5.
12. Sinha, A.K. Colorimetric assay of catalase. *Analytical biochemistry* **1972**, *47*, 389-394, doi:10.1016/0003-2697(72)90132-7.
13. McCord, J.M.; Fridovich, I. Superoxide dismutase. An enzymic function for erythrocuprein (hemocuprein). *The Journal of biological chemistry* **1969**, *244*, 6049-6055.
14. Lowry, O.H.; Rosebrough, N.J.; Farr, A.L.; Randall, R.J. Protein measurement with the Folin phenol reagent. *The Journal of biological chemistry* **1951**, *193*, 265-275.
15. Stepanić, V.; Matijašić, M.; Horvat, T.; Verbanac, D.; Kučerová-Chlupáčová, M.; Saso, L.; Žarković, N. Antioxidant Activities of Alkyl Substituted Pyrazine Derivatives of Chalcones-In Vitro and In Silico Study. *Antioxidants (Basel, Switzerland)* **2019**, *8*, 90, doi:10.3390/antiox8040090.
16. Puntel, G.; de Carvalho, N.; Gubert, P.; Schwertner Palma, A.; Dalla Corte, C.; Avila, D.; Pereira, M.; Gervini, V.; Bresolin, L.; Rocha, J.B., et al. Butane-2,3-dionethiosemicarbazone: An oxime with antioxidant properties. *Chemico-biological interactions* **2008**, *177*, 153-160, doi:10.1016/j.cbi.2008.09.028.

17. Tavadyan, L.A.; Manukyan, Z.H.; Harutyunyan, L.H.; Musayelyan, M.V.; Sahakyan, A.D.; Tonikyan, H.G. Antioxidant Properties of Selenophene, Thiophene and Their Aminocarbonitrile Derivatives. *Antioxidants (Basel, Switzerland)* **2017**, *6*, 22, doi:10.3390/antiox6020022.
18. Sorrenti, V.; Salerno, L.; Giacomo, C.; Acquaviva, R.; Siracusa, M.; Vanella, A. Imidazole derivatives as antioxidants and selective inhibitors of nNOS. *Nitric oxide : biology and chemistry / official journal of the Nitric Oxide Society* **2006**, *14*, 45-50, doi:10.1016/j.niox.2005.09.005.
19. Freire, R.; Morais, S.; Pinheiro, D. Synthesis and antioxidant, anti-inflammatory and gastroprotector activities of anethole and related compounds. *Bioorganic & medicinal chemistry* **2005**, *13*, 4353-4358, doi:10.1016/j.bmc.2005.03.058.
20. Terpinc, P.; Polak, T.; Šegatin, N.; Hanzlowsky, A.; Ulrih, N.; Abramovi, H. Antioxidant properties of 4-vinyl derivatives of hydroxycinnamic acid. *Food Chemistry - FOOD CHEM* **2011**, *128*, 62-69, doi:10.1016/j.foodchem.2011.02.077.
21. Zeliou, K.; Papasotiropoulos, V.; Manoussopoulos, Y.; Lamari, F.N. Physical and chemical quality characteristics and antioxidant properties of strawberry cultivars (Fragaria x ananassa Duch.) in Greece: assessment of their sensory impact. *Journal of the science of food and agriculture* **2018**, *98*, 4065-4073, doi:10.1002/jsfa.8923.
22. Lalitha, P.; Veena, V.; Vidhyapriya, P.; Lakshmi, P.; Krishna, R.; Sakthivel, N. Anticancer potential of pyrrole (1, 2, a) pyrazine 1, 4, dione, hexahydro 3-(2-methyl propyl) (PPDHMP) extracted from a new marine bacterium, Staphylococcus sp. strain MB30. *Apoptosis : an international journal on programmed cell death* **2016**, *21*, 566-577, doi:10.1007/s10495-016-1221-x.
23. Saravana Kumar, P.; Al-Dhabi, N.A.; Duraipandiyan, V.; Balachandran, C.; Praveen Kumar, P.; Ignacimuthu, S. In vitro antimicrobial, antioxidant and cytotoxic properties of Streptomyces lavendulae strain SCA5. *BMC Microbiol* **2014**, *14*, 291-291, doi:10.1186/s12866-014-0291-6.
24. Pinto, M.; AraÚJo, S.; Morais, M.; Sá, N.; Lima, C.; Rosa, C.; Siqueira, E.; Johann, S.; Lima, L. Antifungal and antioxidant activity of fatty acid methyl esters from vegetable oils. *Anais da Academia Brasileira de Ciências* **2017**, *89*, doi:10.1590/0001-3765201720160908.
25. Khan, A.a.; Asuhaimi, F.; Jalal, T.; Roheem, F.; Natto, H.; Farid, J.; Ahmed, Q.; Wahab, R. Hystrix brachyura Bezoar Characterization, Antioxidant Activity Screening, and Anticancer Activity on Melanoma Cells (A375): A Preliminary Study. *Antioxidants* **2019**, *8*, 39, doi:10.3390/antiox8020039.
26. Rahman, M.M.; Ahmad, S.H.; Mohamed, M.T.M.; Ab Rahman, M.Z. Antimicrobial compounds from leaf extracts of Jatropha curcas, Psidium guajava, and Andrographis paniculata. *TheScientificWorldJournal* **2014**, *2014*, 635240-635240, doi:10.1155/2014/635240.
27. Abdel Hady, H.; El-Wakil, E.; Abdel-Gawad, M. GC-MS Analysis, Antioxidant and Cytotoxic Activities of Mentha spicata. *European Journal of Medicinal Plants* **2018**, *26*, 1-12, doi:10.9734/EJMP/2018/45751.
28. Kolak, U.; Gazioğlu, I.; Boga, M.; Özgökçe, F.; Ünal, M.; Choudhary, M.; Ulubelen, A. Phytochemical Investigation of Leontice leontopetalum L. subsp. ewersmannii with Antioxidant and Anticholinesterase Activities. *Records of Natural Products* **2011**, *5*, 309-313.
29. Wiedemann, M.; Gurrola-Diaz, C.M.; Vargas-Guerrero, B.; Wink, M.; Garcia-Lopez, P.M.; Dufer, M. Lupanine Improves Glucose Homeostasis by Influencing KATP Channels and Insulin Gene Expression. *Molecules (Basel, Switzerland)* **2015**, *20*, 19085-19100, doi:10.3390/molecules201019085.
30. Kalpana, D.; R, S.; Mohan, V. GC–MS analysis of ethanol extract of Entada pursaetha DC seed. *Bioscience Discovery* **2012**, *3*, 30-33.
31. Oleszek, M.; Kowalska, I.; Oleszek, W. Phytochemicals in bioenergy crops. *Phytochemistry Reviews* **2019**, *18*, 893-927, doi:10.1007/s11101-019-09639-7.

32. Li, M.; Zhou, L.; Yang, D.; Li, T.; Li, W. Biochemical composition and antioxidant capacity of extracts from Podophyllum hexandrum rhizome. *BMC Complement Altern Med* **2012**, *12*, 263-263, doi:10.1186/1472-6882-12-263.
33. Shareef, H.; Haidar, J.; Hussein, H.; Hameed, I. Antibacterial Effect of Ginger (Zingiber officinale) Roscoe and Bioactive Chemical Analysis using Gas Chromatography Mass Spectrum. *Oriental Journal of Chemistry* **2016**, *32*, 817-837, doi:10.13005/ojc/320207.
34. Erdemoglu, N.; Ozkan, S.; Tosun, F. Alkaloid profile and antimicrobial activity of Lupinus angustifolius L. alkaloid extract. *Phytochemistry Reviews* **2007**, *6*, 197-201, doi:10.1007/s11101-006-9055-8.
35. Yoshida, Y.; Niki, E. Antioxidant Effects of Phytosterol and Its Components. *Journal of nutritional science and vitaminology* **2003**, *49*, 277-280, doi:10.3177/jnsv.49.277.
36. Hailu, Y.; Atlabachew, M.; Chandravanshi, B.; Redi, M. Composition of essential oil and antioxidant activity of Khat (Catha edulis Forsk), Ethiopia. *Chemistry International* **2017**, *3*, 25-31.
37. Alakolanga, A.G.A.W.; Kumar, N.S.; Jayasinghe, L.; Fujimoto, Y. Antioxidant property and [Formula: see text]-glucosidase, [Formula: see text]-amylase and lipase inhibiting activities of Flacourtia inermis fruits: characterization of malic acid as an inhibitor of the enzymes. *J Food Sci Technol* **2015**, *52*, 8383-8388, doi:10.1007/s13197-015-1937-6.
38. Ahmad, M.; Baba, W.N.; Gani, A.; Wani, T.A.; Gani, A.; Masoodi, F.A. Effect of extraction time on antioxidants and bioactive volatile components of green tea (Camellia sinensis), using GC/MS. *Cogent Food & Agriculture* **2015**, *1*, doi:10.1080/23311932.2015.1106387.
39. Meechaona, R.; Sengpracha, W.; Banditpuritat, J.; Kawaree, R.; Phutdhawong, W. Fatty acid content and antioxidant activity of Thai bananas. *Maejo International Journal of Science and Technology* **2007**, *1*, 222-228.
40. Padilla-Gonzalez, G.F.; dos Santos, F.A.; Da Costa, F.B. Sesquiterpene Lactones: More Than Protective Plant Compounds With High Toxicity. *Critical Reviews in Plant Sciences* **2016**, *35*, 18-37, doi:10.1080/07352689.2016.1145956.
41. Mazurek, B.; Chmiel, M.; Górecka, B. Fatty Acids Analysis Using Gas Chromatography-Mass Spectrometer Detector (GC/MSD) - Method Validation Based on Berry Seed Extract Samples. *Food Analytical Methods* **2017**, *10*, 2868-2880, doi:10.1007/s12161-017-0834-1.
42. Rodrigues Ianiski, F.; Bassaco, M.; Vogt, A.; Reis, A.; Pinz, M.; Voss, G.; Oliveira, R.; Silveira, C.; Wilhelm, E.; Luchese, C. Antinociceptive property of vinyl sulfides in spite of their weak antioxidant activity. *Medicinal Chemistry Research* **2017**, *27*, doi:10.1007/s00044-017-2044-1.
43. Sasaki, T.; Yamakoshi, J.; Saito, M.; Kasai, K.; Matsudo, T.; Koga, T.; Mori, K. Antioxidative activities of 4-hydroxy-3(2H)-furanones and their anti-cataract effect on spontaneous cataract rat (ICR/f). *Bioscience, biotechnology, and biochemistry* **1998**, *62*, 1865-1869, doi:10.1271/bbb.62.1865.
44. Rk, M.; Begum, S.; Begum, A.; Koganti, B. Antioxidant potential of piperidine containing compounds - A short review. *Asian Journal of Pharmaceutical and Clinical Research* **2018**, *11*, 66, doi:10.22159/ajpcr.2018.v11i8.26536.
45. Fukai, S.; Tanimoto, S.; Maeda, A.; Fukuda, H.; Okada, Y.; Nomura, M. Pharmacological activity of compounds extracted from persimmon peel (Diospyros kaki THUNB.). *Journal of oleo science* **2009**, *58*, 213-219, doi:10.5650/jos.58.213.
46. Amarowicz, R. Squalene: A natural antioxidant? *European Journal of Lipid Science and Technology* **2009**, *111*, 411-412, doi:10.1002/ejlt.200900102.

47. Caboni, P.; Ntalli, N.; Aissani, N.; Cavoski, I.; Angioni, A. Nematicidal Activity of (E,E)-2,4-Decadienal and (E)-2-Decenal from Ailanthus altissima against Meloidogyne javanica. *Journal of agricultural and food chemistry* **2012**, *60*, 1146-1151, doi:10.1021/jf2044586.
48. Tyagi, T.; Mala, A. Phytochemical and GC-MS analysis of bioactive constituents in the ethanolic of Pistia stratiotes L. and Eichhornia crassipes (Mart.) solms. *Journal of pharmacognosy and phytochemistry* **2017**, *6*, 195-206.
49. Alqahtani, F.Y.; Aleanizy, F.S.; Mahmoud, A.Z.; Farshori, N.N.; Alfaraj, R.; Al-sheddi, E.S.; Alsarra, I.A. Chemical composition and antimicrobial, antioxidant, and anti-inflammatory activities of Lepidium sativum seed oil. *Saudi Journal of Biological Sciences* **2019**, *26*, 1089-1092, doi:https://doi.org/10.1016/j.sjbs.2018.05.007.
50. Said, M.; Ghareb, N.; Hanna, P.; El-Gendy, A. SYNTHESIS AND ANTIOXIDANT ACTIVITY OF NOVEL INDOLE HETEROCYCLES. **2009**.
51. Wei, C.-C.; Yen, P.-L.; Chang, S.-T.; Cheng, P.-L.; Lo, Y.-C.; Liao, V.H.-C. Antioxidative Activities of Both Oleic Acid and Camellia tenuifolia Seed Oil Are Regulated by the Transcription Factor DAF-16/FOXO in Caenorhabditis elegans. *PLoS One* **2016**, *11*, e0157195-e0157195, doi:10.1371/journal.pone.0157195.
52. Hwang, I.G.; Kim, H.Y.; Woo, K.S.; Lee, S.H.; Lee, J.; Jeong, H.S. Isolation and Identification of the Antioxidant DDMP from Heated Pear (Pyrus pyrifolia Nakai). *Prev Nutr Food Sci* **2013**, *18*, 76-79, doi:10.3746/pnf.2013.18.1.076.
53. Luo, Y.; Huang, Y.; Yuan, X.; Zhang, L.; Zhang, X.; Gao, P. Evaluation of Fatty Acid Composition and Antioxidant Activity of Wild-Growing Mushrooms from Southwest China. *International journal of medicinal mushrooms* **2017**, *19*, 937-947, doi:10.1615/IntJMedMushrooms.2017024388.
54. Berdeaux, O.; Voinot, L.; Angioni, E.; Juanéda, P.; Sebedio, j.-l. A simple method of preparation of methyl trans-10,cis-12- and cis-9,trans-11-octadecadienoates from methyl linoleate. *Journal of the American Oil Chemists' Society* **1998**, *75*, 1749-1755, doi:10.1007/s11746-998-0327-x.
55. Shen, C.; Wei, J.; Wang, T.; Wang, Y. Acute toxicity and responses of antioxidant systems to dibutyl phthalate in neonate and adult Daphnia magna. *PeerJ* **2019**, *7*, e6584-e6584, doi:10.7717/peerj.6584.
